# Supplementary material for: Method validation and measurement uncertainty estimation of pesticide residues in Okra by GC/HPLC
Source: PLoS One. 2025 Sep 11;20(9):e0330736. doi: 10.1371/journal.pone.0330736 (PMC12425206; doi:10.1371/journal.pone.0330736)
Supplement: S1 File — (DOCX) [file pone.0330736.s001.docx]

| **Evaluation of bias and precision using reference standard (CRM)** | | | | | | | | | | | | | | |
| --- | --- | --- | --- | --- | --- | --- | --- | --- | --- | --- | --- | --- | --- | --- |
| **Linearity data of Ethion in solvent (n-hexane)** | | | | | | | | | | | | | | |
| **Peak area (mV*min)** | | | | | | | | | | | | | | |
| **True Conc. (mg/kg)** | R1 | | R2 | | R3 | | R4 | R5 | R6 | Mean | | SD | %RSD | |
| 0.05 | 1.3 | | 1.257 | | 1.223 | | 0.92 | 0.99 | 1.335 | 1.171 | | 0.173 | 14.76 | |
| 0.1 | 3.048 | | 2.454 | | 2.6 | | 3.063 | 2.407 | 2.539 | 2.685 | | 0.295 | 10.97 | |
| 0.2 | 5.241 | | 4.844 | | 5.169 | | 4.863 | 5.208 | 5.912 | 5.206 | | 0.387 | 7.44 | |
| 0.5 | 11.319 | | 11.608 | | 11.488 | | 11.162 | 11.461 | 11.5 | 11.423 | | 0.158 | 1.38 | |
| 0.8 | 19.246 | | 18.189 | | 16.96 | | 18.491 | 19.889 | 19.418 | 18.699 | | 1.054 | 5.64 | |
| 1 | 25.288 | | 24.033 | | 23.141 | | 23.889 | 23.088 | 23.595 | 23.839 | | 0.806 | 3.38 | |
| **True Conc.(mg/kg)** | **Calculated Concentration (mg/kg) from peak area**   \|  \| \| --- \| | | | | | | | | | | | | | |
|  | R1 | R2 | | R3 | | R4 | R5 | R6 | Mean | | **SD** | **%RSD/Precision** | | **% Bias** |
| 0.05 | 0.048 | 0.046 | | 0.045 | | 0.032 | 0.035 | 0.049 | 0.042 | | 0.007 | 17.48 | | -15.340 |
| 0.1 | 0.123 | 0.097 | | 0.104 | | 0.123 | 0.095 | 0.101 | 0.107 | | 0.013 | 11.77 | | 7.156 |
| 0.2 | 0.217 | 0.200 | | 0.213 | | 0.200 | 0.215 | 0.245 | 0.215 | | 0.017 | 7.71 | | 7.538 |
| 0.5 | 0.477 | 0.489 | | 0.484 | | 0.470 | 0.483 | 0.485 | 0.481 | | 0.007 | 1.41 | | -3.759 |
| 0.8 | 0.816 | 0.771 | | 0.718 | | 0.784 | 0.844 | 0.823 | 0.793 | | 0.045 | 5.69 | | -0.916 |
| 1 | 1.075 | 1.021 | | 0.983 | | 1.015 | 0.981 | 1.002 | 1.013 | | 0.035 | 3.41 | | 1.271 |

**METHOD VALIDATION PARAMETERS OF ETHION INSECTICIDE (S1)**

**% Bias = (Mean value – Certified value) /Certified value*100 (% Bias <20%) Precision/ % RSD= (Mean of SD / Mean True conc.)*100 (% Precision <20%)**

**Calibration curve of Ethion in solvent (n-hexane)**

(Prepared by) (Checked by) (Approved by)

Dy. QM/TM QM/TM QM/TM

| **True Conc. (mg/kg)** | **Peak Area (mV*min)** | | | | | | | | | | | | | | | | | |
| --- | --- | --- | --- | --- | --- | --- | --- | --- | --- | --- | --- | --- | --- | --- | --- | --- | --- | --- |
|  | R1 | | R2 | | R3 | R4 | R5 | R6 | R7 | R8 | R9 | R10 | **Mean** | | **SD** | | **%RSD** | |
| 0.05 | 1.502 | | 1.473 | | 1.733 | 1.848 | 1.573 | 1.652 |  |  |  |  | 1.630 | | 0.143 | | 8.80 | |
| 0.1 | 2.273 | | 2.757 | | 2.520 | 2.649 | 2.433 | 2.489 |  |  |  |  | 2.520 | | 0.169 | | 6.70 | |
| 0.2 | 6.009 | | 5.912 | | 4.844 | 5.011 | 5.755 | 4.863 |  |  |  |  | 5.399 | | 0.549 | | 10.17 | |
| 0.5 | 13.654 | | 13.854 | | 14.506 | 13.521 | 11.608 | 11.500 | 15.703 | 13.616 | 15.098 | 15.681 | 13.874 | | 1.397 | | 10.07 | |
| 0.8 | 20.633 | | 19.418 | | 21.161 | 21.689 | 18.189 | 18.491 |  |  |  |  | 19.930 | | 1.447 | | 7.26 | |
| 1 | 24.134 | | 24.000 | | 24.187 | 24.505 | 24.036 | 25.688 |  |  |  |  | 24.425 | | 0.644 | | 2.64 | |
| 2 | 47.12 | | 48.621 | | 52.759 | 50.979 | 46.191 | 46.829 |  |  |  |  | 48.750 | | 2.606 | | 5.35 | |
|  | | | | | | | | | | | | | | | | | | |
| **True Conc. (mg/kg)** | |  | | **Calculated Conc. (mg/kg) from peak area** | | | | | | | | | | | | | | |
|  | | R1 | | R2 | R3 | R4 | R5 | R6 | R7 | R8 | R9 | R10 | **Mean** | **SD** | | **% RSD** | | **% Deviation** |
| 0.05 | | 0.036 | | 0.035 | 0.046 | 0.051 | 0.039 | 0.042 |  |  |  |  | 0.042 | 0.006 | | 14.31 | | 16.87 |
| 0.1 | | 0.078 | | 0.088 | 0.078 | 0.084 | 0.084 | 0.077 |  |  |  |  | 0.082 | 0.004 | | 5.42 | | 18.37 |
| 0.2 | | 0.223 | | 0.219 | 0.175 | 0.182 | 0.213 | 0.176 |  |  |  |  | 0.198 | 0.023 | | 11.51 | | 1.06 |
| 0.5 | | 0.540 | | 0.549 | 0.576 | 0.535 | 0.455 | 0.451 | 0.625 | 0.539 | 0.600 | 0.624 | 0.549 | 0.061 | | 11.12 | | -9.88 |
| 0.8 | | 0.830 | | 0.779 | 0.852 | 0.874 | 0.728 | 0.741 |  |  |  |  | 0.801 | 0.060 | | 7.50 | | -0.07 |
| 1 | | 0.975 | | 0.969 | 0.977 | 0.990 | 0.971 | 1.039 |  |  |  |  | 0.987 | 0.027 | | 2.71 | | 1.30 |
| 2 | | 1.928 | | 1.991 | 2.162 | 2.088 | 1.890 | 1.916 |  |  |  |  | 1.996 | 0.108 | | 5.42 | | 0.20 |

**Linearity data of Ethion with matrix (Okra)**

**Matrix effect =** (Slope of matrix curve-slope of solvent curve)/Slope of solvent curve*100 **ME % =**3.11

**Calibration curve of Ethion in matrix (Okra)**

(Prepared by) (Checked by) (Approved by)

Dy. QM/TM QM/TM QM/TM

**Specificity**

.

Chromatogram of control sample in okra with ethion (Rt.= 16.46)

Result: The excipient compounds do not interfere with the analysis of the targeted analyte ethion.

| **Repeatability** | |  |  | **Calculated conc. (mg/kg) from peak area** |
| --- | --- | --- | --- | --- |
| **Replicate** | **Original Conc. (mg/kg)** | **Obtained Rt (min.)** | **Obtained Peak area (mV*min)** |  |
| R1 | 0.5 | 16.44 | 13.654 | 0.54 |
| R2 | 0.5 | 16.46 | 13.854 | 0.549 |
| R3 | 0.5 | 16.465 | 14.506 | 0.576 |
| R4 | 0.5 | 16.383 | 13.521 | 0.535 |
| R5 | 0.5 | 16.472 | 11.608 | 0.455 |
| R6 | 0.5 | 16.427 | 11.5 | 0.451 |
| R7 | 0.5 | 16.464 | 15.703 | 0.625 |
| R8 | 0.5 | 16.482 | 13.616 | 0.539 |
| R9 | 0.5 | 16.447 | 15.098 | 0.6 |
| R10 | 0.5 | 16.430 | 15.681 | 0.624 |
| **Mean** |  | **16.447** | **13.874** | **0.549** |
| **SD** |  | **0.029** | **1.473** | **0.061** |
| **% RSD** |  | **0.018** | **10.614** | **11.113** |

(Prepared by) (Checked by) (Approved by)

Dy. QM/TM QM/TM QM/TM

| **Recovery data of Ethion** | | | | | | | | | | | |
| --- | --- | --- | --- | --- | --- | --- | --- | --- | --- | --- | --- |
| Replicate | **LOQ (0.3 mg/kg)** | | |  | **5 LOQ (1.5 mg/kg)** | | |  | **10 LOQ (3.0 mg/kg)** | | |
|  | Peak area | Conc. (mg/kg) | % Recovery |  | Peak area | Conc. (mg/kg) | % Recovery |  | Peak area | Conc. (mg/kg) | % Recovery |
| **R1** | 6.531 | 0.245 | 81.61 |  | 32.529 | 1.323 | 88.21 |  | 64.75 | 2.660 | 88.65 |
| **R2** | 6.324 | 0.236 | 78.75 |  | 31.85 | 1.295 | 86.33 |  | 64.922 | 2.667 | 88.89 |
| **R3** | 6.449 | 0.241 | 80.48 |  | 30.542 | 1.241 | 82.72 |  | 65.127 | 2.675 | 89.17 |
| **R4** | 6.762 | 0.254 | 84.81 |  | 33.311 | 1.356 | 90.37 |  | 63.114 | 2.592 | 86.39 |
| **R5** | 6.508 | 0.244 | 81.29 |  | 31.937 | 1.299 | 86.57 |  | 61.846 | 2.539 | 84.64 |
| **Mean** | 6.515 | 0.244 | 81.39 |  | 32.034 | 1.303 | 86.84 |  | 63.952 | 2.626 | 87.55 |
| **SD** | 0.160 | 0.007 | 2.209 |  | 1.018 | 0.042 | 2.81 |  | 1.423 | 0.059 | 1.967 |
| **%RSD** | 2.45 | 2.71 | 2.71 |  | 3.18 | 3.24 | 3.24 |  | 2.225 | 2.25 | 2.25 |

| **Calculation of LOD and LOQ** | | **LOD = 3.3 x Residual standard deviation(STEYX) / slope**  **LOQ = 10 x Residual standard deviation (STEYX) / Slope** | | | |
| --- | --- | --- | --- | --- | --- |
| **STEYX :** Standard error of the estimate Y on X through excel | | | | | |
| **True Conc. (mg/kg)** | **Mean area (mV*min)** | | **Steyx value** | **LOD** | **LOQ** |
| 0.05 | 1.630 | | 0.669 | 0.092 | 0.277  Rounded off to 0.3 mg/kg |
| 0.1 | 2.520 | |  |  |  |
| 0.2 | 5.399 | |  |  |  |
| 0.5 | 13.874 | |  |  |  |
| 0.8 | 19.930 | |  |  |  |
| 1 | 24.425 | |  |  |  |

*The obtained LOQ was rounded off to one decimal place

(Prepared by) (Checked by) (Approved by)

Dy. QM/TM QM/TM QM/TM

| **Recovery % of Ethion** | | | | | | | |
| --- | --- | --- | --- | --- | --- | --- | --- |
| **% Recovery** = Average concentration in sample x 100  True Concentration of standard |  |  | LOQ |  | 5 LOQ |  | 10 LOQ |
|  |  | % Recovery | 81.39 |  | 86.84 |  | 87.55 |
|  |  | % RSD | 2.71 |  | 3.24 |  | 2.25 |

Recovery % : Within the recommended range of 70-120% with RSD < 20%

| **Within laboratory reproducibility** | | | | | | | | | |
| --- | --- | --- | --- | --- | --- | --- | --- | --- | --- |
| **Date of analysis 19.12.2023** | | | | | | | | | |
| **Peak Area (mV*min)** | | | | | | | | | |
| **True Conc. (mg/kg)** | R1 | R2 | R3 | R4 | R5 | R6 | Mean | SD | %RSD |
| 0.1 | 2.273 | 2.757 | 2.520 | 2.649 | 2.433 | 2.489 | 2.52 | 0.169 | 6.70 |
| 0.2 | 6.009 | 5.912 | 4.844 | 5.011 | 5.755 | 4.863 | 5.40 | 0.549 | 10.17 |

| **Date of analysis 13.3.2024** | | | | | | | | | |
| --- | --- | --- | --- | --- | --- | --- | --- | --- | --- |
| **Peak Area (mV*min)** | | | | | | | | | |
|  |  |  | | | |  |  |  |  |
| **True Conc. (mg/kg)** | R1 | R2 | R3 | R4 | R5 | R6 | Mean | SD | %RSD |
| 0.1 | 2.76 | 2.550 | 2.645 | 2.534 | 2.776 | 2.76 | 2.671 | 0.110 | 4.13 |
| 0.2 | 5.208 | 5.169 | 5.353 | 5.93 | 5.901 | 5.315 | 5.479 | 0.345 | 6.29 |

Reproducibility : % RSD ≤ 20%

(Prepared by) (Checked by) (Approved by)

Dy. QM/TM QM/TM QM/TM

**Robustness**

| **Changed Carrier gas flow rate : 1.5 ml/min** | | | | | | | | |
| --- | --- | --- | --- | --- | --- | --- | --- | --- |
| **Conc. (mg/kg)** | **Peak area (mV*min)** | | | | | | | |
|  | R1 | R2 | R3 | R4 | R5 | Mean | SD | %RSD |
| 0.2 | 5.253 | 6.418 | 6.426 | 5.395 | 4.856 | 5.670 | 0.715 | 12.61 |

| **Carrier gas flow rate in validated method : 1.2 ml/min** | | | | | | | | |
| --- | --- | --- | --- | --- | --- | --- | --- | --- |
| **Conc. (mg/kg)** | **Peak area (mV*min)** | | | | | | | |
|  | R1 | R2 | R3 | R4 | R5 | Mean | SD | %RSD |
| 0.2 | 6.009 | 5.912 | 4.844 | 5.011 | 5.755 | 5.506 | 0.539 | 9.79 |

| **By change in oven temperature programming** | | | | | | | | | | | | | | | | |
| --- | --- | --- | --- | --- | --- | --- | --- | --- | --- | --- | --- | --- | --- | --- | --- | --- |
| **Oven temperature programming in validated method: 100°C - 180°C @20°C/min. - 270°C @5°C/min. -300°C @13°C/min** | | | | | | | | | | | | | | | | |
| **Conc. (mg/kg)** | | | **Peak area (mV*min)** | | | | |  | | |  | |  |  | |  |
|  | | | R1 | R2 | | | R3 | R4 | | | R5 | | Mean | SD | | %RSD |
| 0.2 | | | 6.009 | 5.912 | | | 4.844 | 5.011 | | | 5.755 | | 5.506 | 0.539 | | 9.79 |
| **Changed oven temperature programming : 60°C - 180°C @20°C/min. - 270°C @5°C/min. -300°C @13°C/min** | | | | | | | | | | | | | | | | |
| **Conc. (mg/kg)** | | **Peak area (mV*min)** | | | | | | |  |  | |  | | |  |  |
|  |  | R1 | | | R2 | R3 | | | R4 | R5 | | Mean | | | SD | %RSD |
| 0.2 | | 5.495 | | | 5.554 | 5.342 | | | 5.496 | 5.118 | | 5.401 | | | 0.177 | 3.27 |

Robustness (RSD ≤ 20%

(Prepared by) (Checked by) (Approved by)

Dy. QM/TM QM/TM QM/TM

Uncertainty measurement of Ethion in Okra

Date of testing: 19.12.2023 T (°C) & RH (%): 25 ± 5

Test parameter:

30-70

Equipment/ Machine used for measurement HPLC


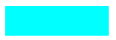


| S. No. | Equip/ Reference Material | Parameter | measured at | Unit |  | u# | Unit |
| --- | --- | --- | --- | --- | --- | --- | --- |
| 1 | CRM-Ethion | Purity | 95.3 | % | u | 1.29 | % |
| 2 | Analytical Balance | Weight | 10 | mg | u | 0.5 | mg |
| 3 | Analytical Balance | Weight | 10 | gm | u | 0.3 | gm |
| 4 | Volumetric Flask | volume | 10 | ml | u | 0.01 | ml |
| 5 | Micropipette | volume | 100 | µl | u | 0.1 | µl |
| 6 | Micropipette | volume | 1000 | µl | u | 0.1 | µl |
| 7 | Recovery | concentration | 86.850 | % |  | 1.258 | % |
| 8 | Linearity | Coefficient of determination |  |  |  | 0.998 |  |

CALCULATIONS

| Uncert. | Source of | Type | Observations Unit | | | | | Deviation Unit | | |  | (Ai-Ᾱ)² | | |  | Std. deviation σ Unit | |
| --- | --- | --- | --- | --- | --- | --- | --- | --- | --- | --- | --- | --- | --- | --- | --- | --- | --- |
| U | uncertainty |  |  | | | | | (Ai-Ᾱ) | | |  |  | | |  | √∑(Ai-Ᾱ)²  = =  √ n-1 0.061  mg/kg | |
| u1 | Repeatabili ty |  | A1 = | | 0.54 0.549 | | mg/kg | -0.009 mg/kg  0.000 mg/kg | | |  | 8.836E-05 1.6E-07 | | |  |  |  |
|  |  |  | A2 = | |  |  | mg/kg |  |  |  |  |  |  |  |  |  |  |
|  |  |  | A3 = | | 0.576 | | mg/kg | 0.027 mg/kg | | |  | 0.0007076 | | |  | Std Uncertainty u1 | |
|  |  |  | A4= | | 0.535 | | mg/kg | -0.014 mg/kg | | |  | 0.0002074 | | |  | = σ/ √n | |
|  |  |  | A5= | | 0.455 | | mg/kg | -0.094 mg/kg | | |  | 0.0089114 | | |  | = 0.01931 mg/kg | |
|  |  |  | A6= | | 0.451 | | mg/kg | -0.098 mg/kg | | |  | 0.0096826 | | |  |  | |
|  |  | A | A7= | | 0.625 | | mg/kg | 0.076 mg/kg | | |  | 0.0001 | | |  | Relative u1 | |
|  |  |  | A8= | | 0.539 | | mg/kg | -0.010 mg/kg | | |  | 0.0001082 | | |  | = 0.01931/0.5494 | |
|  |  |  | A9= | | 0.6 | | mg/kg | 0.051 mg/kg | | |  | 0.0064 | | |  | = 0.03515 | |
|  |  |  | A10= | | 0.624 | | mg/kg | 0.075 mg/kg | | |  | 0.0055652 | | |  |  | |
|  |  |  |  |  | |  | | |  | ∑ = 0.0318 | | |  |  | | |  |
|  |  |  | n = 10 | | | | |  | | |  |  | | |  |  | |
| u2 | CRM- | B | Purity | | | | | Standard Uncertainty | | | u2 | = u/2 | | |  | Relative u2 | |
|  | Ethion |  |  | | | | | ± Uc from Cal Cert | | |  |  | | |  |  | |
|  |  |  | M 95.3 % | | | | | = 1.29 % | | | = | 0.645 % | | |  | = 0.00677 | |
|  |  |  |  | | | | | at k = 2 | | |  |  | | |  |  | |
| u3 | Analytical | B | Weight | | | | | Standard Uncertainty | | | u3 | = u/2 | | |  | Relative u3 | |
|  | Balance |  |  | | | | | ± Uc from Cal Cert | | |  |  | | |  |  | |
|  |  |  | M 10 mg | | | | | = 0.5 mg | | | = | 0.25 mg | | |  | = 0.02500 | |
|  |  |  |  | | | | | at k = 2 | | |  |  | | |  |  | |
| u4 | Analytical | B | Weight | | | | | Standard Uncertainty | | | u4 | = u/2 | | |  | Relative u4 | |
|  | Balance |  |  | | | | | ± Uc from Cal Cert | | |  |  | | |  |  | |
|  |  |  | M 10 gm | | | | | = 0.3 gm | | | = | 0.15 gm | | |  | = 0.01500 | |
|  |  |  |  | | | | | at k = 2 | | |  |  | | |  |  | |
| u5 | Volumetric | B | volume | | | | | Standard Uncertainty | | | u5 | = u/2 | | |  | Relative u5 | |
|  | flask |  |  | | | | | ± Uc from Cal Cert | | |  |  | | |  |  | |
|  |  |  | M 10 ml | | | | | = 0.01 µl | | | = | 0.005 µl | | |  | = 0.00050 | |
|  |  |  |  | | | | | at k = 2 | | |  |  | | |  |  | |
| u6 | Micropipet | B | volume | | | | | Standard Uncertainty | | | u6 | = u/2 | | |  | Relative u6 | |
|  | te |  |  | | | | | ± Uc from Cal Cert | | |  |  | | |  |  | |
|  |  |  | M 100 µl | | | | | = 0.1 µl | | | = | 0.05 µl | | |  | = 0.00050 | |
|  |  |  |  | | | | | at k = 2 | | |  |  | | |  |  | |
| u7 | Micropipet | B | volume | | | | | Standard Uncertainty | | | u7 | = u/2 | | |  | Relative u7 | |
|  | te |  |  | | | | | ± Uc from Cal Cert | | |  |  | | |  |  | |
|  |  |  | M 1000 µl | | | | | = 0.1 µl | | | = | 0.05 µl | | |  | = 0.00005 | |
|  |  |  |  | | | | | at k = 2 | | |  |  | | |  |  | |

| u8 | Recovery | B | concentration |  | Standard Uncertainty |  | u8 = uc/√3 | Relative u8 |
| --- | --- | --- | --- | --- | --- | --- | --- | --- |
|  |  |  |  |  | ±Uc from recovery |  |  |  |
|  |  |  | M 86.85 % |  | = 1.258 | % | = 0.73 % | = 0.0084 |
|  |  |  |  |  |  |  |  |  |
| u9 | Linearity | B | Coefficient of determination |  | Standard Uncertainty |  | u9 = uc/√3 | Relative u9 |
|  |  |  |  |  | ±Uc from linearity curve |  |  |  |
|  |  |  | M 1 |  | = 0.002 |  | = 0.001154 | = 0.0012 |
|  |  |  |  |  |  |  |  |  |

Uncertainty Budget

| Uncert. | Source of Uncertaint y | Estimate Value | Limits | Type | Distribution | F | Std. Uncertainty | Sensi-tivity co-efficient | Uncert. Contribu-  tion | DOF |
| --- | --- | --- | --- | --- | --- | --- | --- | --- | --- | --- |
| u1 | Repeatabili ty | 0.061 | 0.030525 | A | Normal | √10 | 0.019305705 | 1 | 0.03515 | 9 |
| u2 | CRM- Ethion | 1.29 | 0.645 | B | Normal | 2 | 0.00677 | 1 | 0.00677 | ∞ |
| u3 | Analytical Balance | 0.5 | 0.25 | B | Normal | 2 | 0.025 | 1 | 0.025 | ∞ |
| u4 | Analytical Balance | 0.3 | 0.15 | B | Normal | 2 | 0.015 | 1 | 0.015 | ∞ |
| u5 | Volumetric flask | 0.01 | 0.005 | B | Normal | 2 | 0.0005 | 1 | 0.0005 | ∞ |
| u6 | Micropipet te | 0.1 | 0.05 | B | Normal | 2 | 0.0005 | 1 | 0.0005 | ∞ |
| u7 | Micropipet te | 0.1 | 0.05 | B | Normal | 2 | 0.00005 | 1 | 0.00005 | ∞ |
| u8 | Recovery | 1.258 | 0.629 | B | Rectangular | √3 | 0.0084 | 1 | 0.0084 | ∞ |
| u9 | Linearity | 0.002 | 0.001 | B | Rectangular | √3 | 0.0012 | 1 | 0.0012 | ∞ |

Combined Rel. Std. Uc = √{(u1)² + (u2)² + (u3)² + (u4)² + (u5)² + (u6)² + (u7)² + (u8)² + (u9)²}

Uc = √{(0.03515)² + (0.00677)² + (0.025)² + (0.015)² + (0.0005)² + (0.0005)² + (0.00005)² + (0.0084)² + (0.0012)²} Uc = 0.047

Degree of Freedom (DoF) =


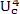

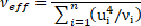


- (0.047^4)/{(0.03515^4/9)+(0.00677^4/∞)+(0.025^4/∞)+(0.015^4/∞)+(0.0005^4/∞)+(0.0005^4/∞)+(0.00005^4/∞)+(0.0084^4/∞)+(0.0012^4/∞)}
- (0.047^4)/((0.03515^4)/9)= 28.8

Coverage factor k at 95% Confidence Level = 2.05 (from student t table for DoF)

Expanded Uncertainty for mean 0.549 mg/kg , UM = 0.047 x 2.05 x 0.549 = 0.053 mg/kg

(Prepared by ) (Checked by) (Approved by)
