# Supplementary material for: Method validation and measurement uncertainty estimation of pesticide residues in Okra by GC/HPLC
Source: PLoS One. 2025 Sep 11;20(9):e0330736. doi: 10.1371/journal.pone.0330736 (PMC12425206; doi:10.1371/journal.pone.0330736)
Supplement: S2 File — (DOCX) [file pone.0330736.s002.docx]

**METHOD VALIDATION PARAMETERS OF LAMBDA CYHALOTHRIN INSECTICIDE (S2)**

**Evaluation of Bias and Precision using reference standard (CRM)**

| **Linearity data of lambda Cyhalothrin in solvent (n-hexane)** | | | | | | | | | | | | | |
| --- | --- | --- | --- | --- | --- | --- | --- | --- | --- | --- | --- | --- | --- |
| **Peak Area (mV*min)** | | | | | | | | | | | | | |
| **True Conc. (mg/Kg)** | **R1** | **R2** | **R3** | **R4** | **R5** | **R6** | | **Mean** | | **SD** | | **% RSD** | |
| 0.05 | 0.557 | 0.584 | 0.512 | 0.648 | 0.49 | 0.52 | | 0.552 | | 0.058 | | 10.48 | |
| 0.1 | 1.187 | 1.173 | 1.295 | 1.363 | 1.183 | 1.35 | | 1.259 | | 0.088 | | 6.99 | |
| 0.2 | 2.154 | 2.567 | 2.176 | 2.347 | 2.449 | 2.106 | | 2.300 | | 0.184 | | 8.02 | |
| 0.5 | 5.487 | 5.259 | 5.73 | 5.502 | 5.184 | 5.627 | | 5.465 | | 0.210 | | 3.84 | |
| 1 | 12.054 | 11.551 | 11.807 | 12.349 | 11.066 | 11.996 | | 11.804 | | 0.448 | | 3.80 | |
| 2 | 24.799 | 25.059 | 22.976 | 22.768 | 23.309 | 24.923 | | 23.972 | | 1.063 | | 4.43 | |
| **Calculated Concentration (mg/Kg) from peak area** | | | | | | | | | | | | | |
| **True Conc. (mg/Kg)** | **R1** | **R2** | **R3** | **R4** | **R5** | **R6** | **Mean** | | **SD** | | **% RSD/ Precisison** | | **% Bias** |
| 0.05 | 0.058 | 0.061 | 0.055 | 0.066 | 0.053 | 0.055 | 0.058 | | 0.004 | | 7.60 | | 15.81 |
| 0.1 | 0.111 | 0.110 | 0.120 | 0.126 | 0.111 | 0.124 | 0.113 | | 0.007 | | 6.01 | | 13.44 |
| 0.2 | 0.191 | 0.226 | 0.193 | 0.208 | 0.216 | 0.187 | 0.204 | | 0.014 | | 6.89 | | 1.75 |
| 0.5 | 0.469 | 0.450 | 0.489 | 0.470 | 0.444 | 0.481 | 0.470 | | 0.016 | | 3.40 | | -6.08 |
| 1 | 1.016 | 0.975 | 0.996 | 1.041 | 0.934 | 1.012 | 0.996 | | 0.034 | | 3.43 | | -0.44 |
| 2 | 2.079 | 2.100 | 1.927 | 1.909 | 1.954 | 2.089 | 2.035 | | 0.081 | | 4.00 | | 1.75 |

**% Bias** = (Mean Value – Certified Value)/ Certified value *100 (% Bias <20%); **Precision / % RSD** = Mean of SD/ Mean True Conc)*100 (% Precision <20%)

**Calibration curve of Lambda Cyhalothrin in solvent (n-hexane)**

**Linearity data of Lambda Cyhalothrin with Matrix (Okra)**

| **Peak Area (mV*min)** | | | | | | | | | | | | | | | | | | | | | | | | |  | |
| --- | --- | --- | --- | --- | --- | --- | --- | --- | --- | --- | --- | --- | --- | --- | --- | --- | --- | --- | --- | --- | --- | --- | --- | --- | --- | --- |
| **True Conc. (mg/Kg)** | | **R1** | | **R2** | | **R3** | | **R4** | | **R5** | | **R6** | | **R7** | | **R8** | | **R9** | | **R10** | | **Mean** | | **SD** | **% RSD** | |
| 0.05 | | 0.651 | | 0.658 | | 0.603 | | 0.632 | | 0.685 | | 0.656 | |  | |  | |  | |  | | 0.028 | | 4.27 | 0.651 | |
| 0.1 | | 1.375 | | 1.370 | | 1.223 | | 1.277 | | 1.365 | | 1.340 | |  | |  | |  | |  | | 0.775 | | 58.46 | 1.375 | |
| 0.2 | | 2.547 | | 2.595 | | 2.106 | | 2.243 | | 2.317 | | 2.435 | |  | |  | |  | |  | | 0.507 | | 21.35 | 2.547 | |
| 0.5 | | 5.982 | | 6.032 | | 6.054 | | 6.071 | | 6.049 | | 5.996 | | 6.073 | | 5.713 | | 5.665 | | 5.845 | | 0.152 | | 2.56 | 5.982 | |
| 1 | | 12.731 | | 12.884 | | 12.316 | | 12.327 | | 11.506 | | 11.943 | |  | |  | |  | |  | | 0.187 | | 1.52 | 12.731 | |
| 2 | | 26.543 | | 26.044 | | 25.879 | | 25.877 | | 24.258 | | 25.528 | |  | |  | |  | |  | | 0.062 | | 0.24 | 26.543 | |
| **Calculated Concentration (mg/Kg) from peak area** | | | | | | | | | | | | | | | | | | | | | | | | | | |
| **True Conc. (mg/Kg)** | **R1** | | **R2** | | **R3** | | **R4** | | **R5** | | **R6** | | **R7** | | **R8** | | **R9** | | **R10** | | **Mean** | | **SD** | **% RSD** | | **% Deviation** |
| 0.05 | 0.056 | | 0.056 | | 0.052 | | 0.054 | | 0.058 | | 0.056 | |  | |  | |  | |  | | 0.055 | | 0.002 | 3.97 | | -10.84 |
| 0.1 | 0.113 | | 0.113 | | 0.101 | | 0.106 | | 0.113 | | 0.111 | |  | |  | |  | |  | | 0.109 | | 0.005 | 4.50 | | -9.40 |
| 0.2 | 0.207 | | 0.211 | | 0.172 | | 0.183 | | 0.188 | | 0.198 | |  | |  | |  | |  | | 0.193 | | 0.015 | 7.72 | | 3.51 |
| 0.5 | 0.480 | | 0.484 | | 0.486 | | 0.488 | | 0.486 | | 0.482 | | 0.488 | | 0.459 | | 0.455 | | 0.470 | | 0.478 | | 0.012 | 2.54 | | 4.45 |
| 1 | 1.018 | | 1.030 | | 0.985 | | 0.986 | | 0.921 | | 0.955 | |  | |  | |  | |  | | 0.983 | | 0.040 | 4.11 | | 1.73 |
| 2 | 2.119 | | 2.079 | | 2.066 | | 2.066 | | 1.937 | | 2.038 | |  | |  | |  | |  | | 2.051 | | 0.062 | 3.01 | | -2.53 |

**Matrix Effect** = (Slope of Matrix Curve-Slope of Solvent Cure)/ Slope of Solvent Curve*100 ME % = 4.38

**Calibration curve of Lambda Cyhalothrin in matrix (Okra)**

**Specificity**

Chromatogram of control sample in okra with Lambda Cyhalothrin (Rt = 21.48 min)

Result: The excipient compound do not interfere with the analysis of the targeted analyte Lambda Cyhalothrin

**Repeatability**

| **Replicates** | **Original Conc. (mg/Kg)** | **Obtained Retention Time (Min)** | **Obtained Peak Area (mV*min)** | **Calculate Conc. (mg/Kg) from Peak area** |
| --- | --- | --- | --- | --- |
| **R1** | 0.5 | 21.485 | 5.982 | 0.48 |
| **R2** | 0.5 | 21.485 | 6.032 | 0.484 |
| **R3** | 0.5 | 21.478 | 6.054 | 0.486 |
| **R4** | 0.5 | 21.495 | 6.071 | 0.488 |
| **R5** | 0.5 | 21.483 | 6.049 | 0.486 |
| **R6** | 0.5 | 21.475 | 5.996 | 0.482 |
| **R7** | 0.5 | 21.487 | 6.073 | 0.488 |
| **R8** | 0.5 | 21.493 | 5.713 | 0.459 |
| **R9** | 0.5 | 21.465 | 5.665 | 0.455 |
| **R10** | 0.5 | 21.493 | 5.845 | 0.47 |
| **Mean** |  | 21.48 | 5.948 | 0.478 |
| **SD** |  | 0.009 | 0.152 | 0.012 |
| **% RSD** |  | 0.04 | 2.56 | 2.55 |

**Limit of Detection (LOD) and Limit of Quantification (LOQ)**

| **LOD =** 3.3 x Residual standard deviation (STEYX) / Slope | | | | |
| --- | --- | --- | --- | --- |
| **LOQ =** 10 x Residual standard deviation (STEYX) / Slope | | | | |
| **STEYX =** Standard Error of the estimate Y on X through excel | | | | |
| **True Conc. (mg/Kg)** | **Mean Area (mAU*min)** | **Steyx Value** | **LOD** | **LOQ** |
| 0.05 | 0.648 | 0.346 | 0.091 | 0.275  Rounded off to 0.3 mg/Kg |
| 0.1 | 1.325 |  |  |  |
| 0.2 | 2.374 |  |  |  |
| 0.5 | 5.948 |  |  |  |
| 1 | 12.285 |  |  |  |
| 2 | 25.688 |  |  |  |
|  | 49.948 |  |  |  |

**Recovery of Lambda Cyhalothrin**

| **Replicate** | **LOQ (0.3 mg/Kg)** | | | **5 LOQ (1.5 mg/Kg)** | | | **10 LOQ (3 mg/Kg)** | | |
| --- | --- | --- | --- | --- | --- | --- | --- | --- | --- |
|  | **Peak Area** | **Conc (mg/Kg)** | **% Recovery** | **Peak Area** | **Conc (mg/Kg)** | **% Recovery** | **Peak Area** | **Conc (mg/Kg)** | **% Recovery** |
| **R1** | 3.088 | 0.250 | 83.29 | 17.035 | 1.36 | 90.75 | 34.985 | 2.791 | 93.05 |
| **R2** | 3.118 | 0.252 | 84.09 | 16.604 | 1.33 | 88.46 | 35.123 | 2.802 | 93.42 |
| **R3** | 2.924 | 0.237 | 78.94 | 16.842 | 1.35 | 89.72 | 34.358 | 2.742 | 91.38 |
| **R4** | 2.98 | 0.241 | 80.42 | 16.038 | 1.28 | 85.45 | 35.657 | 2.845 | 94.83 |
| **R5** | 3.069 | 0.248 | 82.79 | 16.873 | 1.35 | 89.89 | 36.674 | 2.926 | 97.54 |
| **Mean** | 3.036 | 0.246 | 81.91 | 16.678 | 1.33 | 88.85 | 35.359 | 2.821 | 94.04 |
| **SD** | 0.081 | 0.006 | 2.15 | 0.390 | 0.031 | 2.070 | 0.868 | 0.069 | 2.306 |
| **% RSD** | 2.67 | 2.62 | 2.62 | 2.34 | 2.33 | 2.33 | 2.46 | 2.45 | 2.45 |

**Percent Recovery**

| **% Recovery** = Average Concentration in sample x 100  True Concentration in sample | | | |
| --- | --- | --- | --- |
|  | **LOQ** | **5 LOQ** | **10 LOQ** |
| **% Recovery** | 81.91 | 88.85 | 94.04 |
| **% RSD** | 2.62 | 2.33 | 2.45 |

Recovery % - Within the recommended range of 70-120% with RSD <20%

**Reproducibility of Lambda Cyhalothrin**

| **Within Laboratory** | | | | | | | | | |
| --- | --- | --- | --- | --- | --- | --- | --- | --- | --- |
| **Date of Analysis: 7.12.2023** | | | | | | | | | |
| **True Conc. (mg/Kg)** | **Peak Area (mV*min)** | | | | | | **Mean** | **SD** | **% RSD** |
|  | **R1** | **R2** | **R3** | **R4** | **R5** | **R6** |  |  |  |
| 0.2 | 2.547 | 2.595 | 2.106 | 2.243 | 2.317 | 2.435 | 2.374 | 0.187 | 7.87 |
| 0.5 | 5.982 | 6.032 | 6.054 | 6.071 | 6.049 | 5.996 | 6.031 | 0.035 | 0.58 |
|  | | | | | | | | | |
| **Date of Analysis: 15.3.2024** | | | | | | | | | |
| **True Conc. (mg/Kg)** | **Peak Area (mV*min)** | | | | | | **Mean** | **SD** | **% RSD** |
|  | **R1** | **R2** | **R3** | **R4** | **R5** | **R6** |  |  |  |
| 0.2 | 2.688 | 2.719 | 2.449 | 2.904 | 2.687 | 2.887 | 2.722 | 0.166 | 6.09 |
| 0.5 | 6.02 | 5.748 | 5.749 | 5.443 | 5.479 | 5.725 | 5.694 | 0.211 | 3.70 |

Reproducibility RSD < 20%

**Robustness of Lambda Cyhalothrin**

| **Carrier gas flow rate in validated method: 1.2 ml/min** | | | | | | | | |
| --- | --- | --- | --- | --- | --- | --- | --- | --- |
| **Peak Area (mV*min)** | | | | | | | | |
| **True Conc. (mg/Kg)** | **R1** | **R2** | **R3** | **R4** | **R5** | **Mean** | **SD** | **% RSD** |
| 0.2 | 2.547 | 2.595 | 2.106 | 2.243 | 2.317 | 2.362 | 0.206 | 8.74 |
|  | | | | | | | | |
| **Carrier gas flow rate in changed method: 1.5 ml/min** | | | | | | | | |
| **True Conc. (mg/Kg)** | **R1** | **R2** | **R3** | **R4** | **R5** | **Mean** | **SD** | **% RSD** |
| 0.2 | 2.532 | 2.607 | 2.575 | 2.201 | 2.566 | 2.496 | 0.167 | 6.70 |

| **Oven temperature programming : 100°C - 180°C @20°C/min. - 270°C @5°C/min. -300°C @13°C/min in validated method:** | | | | | | | | |
| --- | --- | --- | --- | --- | --- | --- | --- | --- |
| **Peak Area (mV*min)** | | | | | | | | |
| **True Conc. (mg/Kg)** | **R1** | **R2** | **R3** | **R4** | **R5** | **Mean** | **SD** | **% RSD** |
| 0.2 | 2.547 | 2.595 | 2.106 | 2.243 | 2.317 | 2.362 | 0.206 | 8.74 |
|  | | | | | | | | |
| **Oven temperature programming in changed method: 60°C - 180°C @20°C/min. - 270°C @5°C/min. -300°C @13°C/min** | | | | | | | | |
| **True Conc. (mg/Kg)** | **R1** | **R2** | **R3** | **R4** | **R5** | **Mean** | **SD** | **% RSD** |
| 0.2 | 2.56 | 2.589 | 2.619 | 2.475 | 2.547 | 2.558 | 0.054 | 2.11 |

Robustness RSD < 20%

**Uncertainty measurement of lamba Cyhalothrin in Okra**

Test parameter : lamba Cyhalothrin Product : Okra Date of testing : 7.12.2023

Equipment Machine used for measurement GC-ECD Temp. (ºC) : 25±5

RH (%) : 30-70

| S.NO. | Equip/Referencr Material | Parameter | Measured at | Unit | U# | Unit |
| --- | --- | --- | --- | --- | --- | --- |
| 1 | CRM-lamba Cyhalothrin | Purity | 99.9 | % | u | 0.8 |
| 2 | Analytical Balance | Weight | 10 | mg | u | 0.5 |
| 3 | Analytical Balance | weight | 10 | gm | u | 0.3 |
| 4 | Volumetric Flask | volume | 10 | ml | u | 0.01 |
| 5 | Micropipette | volume | 100 | µl | u | 0.1 |
| 6 | Micropipette | volume | 1000 | µl | u | 0.1 |
| 7 | Recovery | Concentration | 88.85 | % |  | 0.926 |
| 8 | Linearity | Coefficient of determination |  |  |  | 0.999 |

CALCULATIONS

| Uncert  U | Source of uncertainty | Type | Observations | | Unit | Deviation  (Ai-Ᾱ) | Unit | (Ai-A)^2^ | Std. deviation Unit |
| --- | --- | --- | --- | --- | --- | --- | --- | --- | --- |
| u 1 | Repeatabiliy | A | A1= | 0.48 | mg/kg | 0.002 | mg/kg | 4.84E-06 | \| Std. deviation σ \| \| Unit \| \| --- \| --- \| --- \| \| = \| √∑(Ai-Ᾱ)² \|  \| \| √ n-1 \|  \| \| = \| 0.012 \| mg/Kg \| \| Std Uncertainty u1 \| \|  \| \| = \| σ/ √n \|  \| \| = \| 0.00385 \| mg/Kg \| \|  \|  \|  \| \| Relative u1 \| \|  \| \| = \| 0.00385/0.4778 \| \| \| = \| 0.00806 \|  \| |
|  |  |  | A2= | 0.484 | mg/kg | 0.006 | mg/kg | 3.84E-05 |  |
|  |  |  | A3= | 0.486 | mg/kg | 0.008 | mg/kg | 6.72E-05 |  |
|  |  |  | A4= | 0.488 | mg/kg | 0.010 | mg/kg | 0.000104 |  |
|  |  |  | A5= | 0.486 | mg/kg | 0.008 | mg/kg | 6.72E-05 |  |
|  |  |  | A6= | 0.482 | mg/kg | 0.004 | mg/kg | 1.76E-05 |  |
|  |  |  | A7= | 0.488 | mg/kg | 0.010 | mg/kg | 0.0001 |  |
|  |  |  | A8= | 0.459 | mg/kg | -0.019 | mg/kg | 0.000353 |  |
|  |  |  | A9= | 0.455 | mg/kg | -0.023 | mg/kg | 0.0064 |  |
|  |  |  | A10= | 0.47 | mg/kg | -0.008 | mg/kg | 6.08E-05 |  |
|  |  |  | Mean = 0.478  N= 10 | | mg/kg |  | | ∑ = 0.0072 |  |
| u 2 | CRM-lamda Cyhalothrin | B | Purity 99.9% | | | Standard uncertainty  ±Uc from Cal. Cert.  = 0.8%  At k=2 | | u 2 / 2 =   0.4% | Relative u 2  = 0.00400 |
| u 3 | Analytical Balance | B | Weight 10 mg | | | Standard uncertainty  ±Uc from Cal. Cert.  = 0.5 mg  At k= 2 | | u 3 / 2 =  0.025 mg | Relative u 3  = 0.02500 |
| u 4 | Analytical Balance | B | Weight 10 gram | | | Standard uncertainty  ±Uc from Cal. Cert.  = 0.3 gm  At k=2 | | u 4 / 2 =  0.15 gm | Relative u 4  = 0.001500 |
| u 5 | Volumetric flask | B | Volume 10 ml | | | Standard uncertainty  ±Uc from Cal. Cert.  = 0.01 ml  At k= 2 | | u 5 / 2 =  0.005 ml | Relative u 5  = 0.00050 |
| u 6 | Micropipette | B | Volume 100 µl | | | Standard uncertainty  ±Uc from Cal. Cert.  = 0.1µl  At k= 2 | | u 6 / 2 =  0.05 µl | Relative u 6  = 0.00050 |
| u 7 | Micropipette | B | Volume 1000 µl | | | Standard uncertainty  ±Uc from Cal. Cert.  = 0.1µl  At k= 2 | | u 7 / 2 =  0.05 µl | Relative u 7  = 0.00005 |
| u 8 | Recovery | B | Concentration 88.85 % | | | Standard uncertainty  ±Uc from recovery.  = 0.926%  At k= 2 | | u 8 / √3 =  0.53 % | Relative u 8  = 0.006 |
| u 9 | Linearity | B | Coefficient of variation = 1 | | | Standard uncertainty  ±Uc from linearity curve  = 0.001  At k= 2 | | u 9 / √3 =  0.000577 | Relative u 9  = 0.0006 |

**Uncertainty Budget**

| Uncert | Source of Uncertainty | Estimate Value | Limits | Type | Distribution | F | Std. Uncertainty | Sensi-tivity co-efficient | Uncertainty contribution | D O F |
| --- | --- | --- | --- | --- | --- | --- | --- | --- | --- | --- |
| u 1 | Repeatability | 0.012 | 0.006095 | A | Normal | √10 | 0.003854816 | 1 | 0.00806 | 9 |
| u 2 | CRM-lambda Cyhalothrin | 0.8 | 0.4 | B | Normal | 2 | 0.004 | 1 | 0.004 | ∞ |
| u 3 | Analytical Balance | 0.5 | 0.25 | B | Normal | 2 | 0.025 | 1 | 0.025 | ∞ |
| u 4 | Analytical Balance | 0.3 | 0.15 | B | Normal | 2 | 0.015 | 1 | 0.015 | ∞ |
| u 5 | Volumetric Flask | 0.01 | 0.005 | B | Normal | 2 | 0.0005 | 1 | 0.0005 | ∞ |
| u 6 | Micropipette | 0.1 | 0.05 | B | Normal | 2 | 0.0005 | 1 | 0.0005 | ∞ |
| u 7 | Micropipette | 0.1 | 0.05 | B | Normal | 2 | 0.00005 | 1 | 0.00005 | ∞ |
| u 8 | Recovery | 0.926 | 0.463 | B | Rectangular | √3 | 0.006 | 1 | 0.006 | ∞ |
| u 9 | Linearity | 0.001 | 0.0005 | B | Rectangular | √3 | 0.0006 | 1 | 0.0006 | ∞ |

Combined Rel. Std. Uc = √{(u1)² + (u2)² + (u3)² + (u4)² + (u5)² + (u6)² + (u7)² + (u8)² + (u9)²}

Uc = √{(0.00806)² + (0.004)² + (0.025)² + (0.015)² + (0.0005)² + (0.0005)² + (0.00005)² + (0.006)² + (0.0006)²}

Uc = 0.031


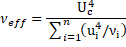


Degree of Freedom (DOF) =

=(0.031^4)/{(0.00806^4/9)+(0.004^4/∞)+(0.025^4/∞)+(0.015^4/∞)+(0.0005^4/∞)+(0.0005^4/∞)+(0.00005^4/∞)+(0.006^4/∞)+(0.0006^4/∞)}

= (0.031^4)/((0.00806^4)/9)= 1969.5

Coverage factor k at 95% Confidence Level = 1.96 (from student t table for DoF)

**Expanded Uncertainty for mean 0.478 mg/kg, UM = 0.031 x 1.96 x 0.478 = 0.029 mg/kg**
