# Supplementary material for: Method validation and measurement uncertainty estimation of pesticide residues in Okra by GC/HPLC
Source: PLoS One. 2025 Sep 11;20(9):e0330736. doi: 10.1371/journal.pone.0330736 (PMC12425206; doi:10.1371/journal.pone.0330736)
Supplement: S3 File — (DOCX) [file pone.0330736.s003.docx]

**METHOD VALIDATION PARAMETERS OF THIAMETHOXAM INSECTICIDE (S3)**

**Evaluation of Bias and Precision using reference standard (CRM)**

| **Linearity data of Thiamethoxam in solvent (Acetonitrile)** | | | | | | | | | | | | | |
| --- | --- | --- | --- | --- | --- | --- | --- | --- | --- | --- | --- | --- | --- |
| **Peak Area (mAU*min)** | | | | | | | | | | | | | |
| **True Conc. (mg/Kg)** | **R1** | **R2** | **R3** | **R4** | **R5** | **R6** | | **Mean** | | **SD** | | **% RSD** | |
| 0.1 | 0.205 | 0.208 | 0.199 | 0.197 | 0.206 | 0.199 | | 0.202 | | 0.005 | | 2.25 | |
| 0.2 | 0.385 | 0.383 | 0.419 | 0.425 | 0.423 | 0.42 | | 0.409 | | 0.020 | | 4.80 | |
| 0.5 | 1.203 | 1.176 | 1.218 | 1.248 | 1.102 | 1.1 | | 1.175 | | 0.062 | | 5.24 | |
| 1 | 2.273 | 2.325 | 2.303 | 2.251 | 2.253 | 2.254 | | 2.277 | | 0.031 | | 1.36 | |
| 2 | 4.587 | 4.616 | 4.4 | 4.322 | 4.343 | 4.39 | | 4.443 | | 0.126 | | 2.85 | |
| 5 | 11.509 | 11.61 | 11.381 | 11.3 | 11.414 | 11.398 | | 11.435 | | 0.109 | | 0.95 | |
| **Calculated Concentration (mg/Kg) from peak area** | | | | | | | | | | | | | |
| **True Conc. (mg/Kg)** | **R1** | **R2** | **R3** | **R4** | **R5** | **R6** | **Mean** | | **SD** | | **% RSD/ Precisison** | | **% Bias** |
| 0.1 | 0.103 | 0.104 | 0.100 | 0.099 | 0.103 | 0.100 | 0.102 | | 0.002 | | 1.90 | | 2.32 |
| 0.2 | 0.181 | 0.181 | 0.196 | 0.199 | 0.198 | 0.197 | 0.186 | | 0.008 | | 4.07 | | -6.94 |
| 0.5 | 0.539 | 0.527 | 0.546 | 0.559 | 0.495 | 0.494 | 0.537 | | 0.027 | | 4.98 | | 7.48 |
| 1 | 1.007 | 1.030 | 1.020 | 0.997 | 0.998 | 0.999 | 1.019 | | 0.014 | | 1.38 | | 1.89 |
| 2 | 2.019 | 2.031 | 1.937 | 1.903 | 1.912 | 1.933 | 1.996 | | 0.051 | | 2.54 | | -0.21 |
| 5 | 5.045 | 5.090 | 4.990 | 4.954 | 5.004 | 4.997 | 5.042 | | 0.047 | | 0.93 | | 0.83 |

**% Bias** = (Mean Value – Certified Value)/ Certified value *100 (% Bias <20%); **Precision / % RSD** = Mean of SD/ Mean True Conc)*100 (% Precision <20%)

**Calibration curve of Thiamethoxam n solvent (Acetonitrile)**

**Linearity data of Thiamethoxam with Matrix (Okra)**

| **Peak Area (mAU*min)** | | | | | | | | | | | | | | | | | | | | | | | | |  | |
| --- | --- | --- | --- | --- | --- | --- | --- | --- | --- | --- | --- | --- | --- | --- | --- | --- | --- | --- | --- | --- | --- | --- | --- | --- | --- | --- |
| **True Conc. (mg/Kg)** | | **R1** | | **R2** | | **R3** | | **R4** | | **R5** | | **R6** | | **R7** | | **R8** | | **R9** | | **R10** | | **Mean** | | **SD** | **% RSD** | |
| 0.1 | | 0.262 | | 0.305 | | 0.302 | | 0.303 | | 0.306 | | 0.251 | |  | |  | |  | |  | | 0.288 | | 0.025 | 8.61 | |
| 0.2 | | 0.459 | | 0.48 | | 0.451 | | 0.464 | | 0.436 | | 0.449 | |  | |  | |  | |  | | 0.457 | | 0.015 | 3.28 | |
| 0.5 | | 1.253 | | 1.27 | | 1.207 | | 1.276 | | 1.292 | | 1.294 | | 1.124 | | 1.292 | | 1.106 | | 1.103 | | 1.222 | | 0.081 | 6.61 | |
| 1 | | 2.418 | | 2.480 | | 2.244 | | 2.334 | | 2.346 | | 2.346 | |  | |  | |  | |  | | 2.361 | | 0.080 | 3.40 | |
| 2 | | 4.553 | | 4.507 | | 4.54 | | 4.565 | | 4.564 | | 4.742 | |  | |  | |  | |  | | 4.579 | | 0.083 | 1.81 | |
| 5 | | 11.259 | | 11.965 | | 11.845 | | 11.893 | | 11.823 | | 11.416 | |  | |  | |  | |  | | 11.700 | | 0.289 | 2.47 | |
| **Calculated Concentration (mg/Kg) from peak area** | | | | | | | | | | | | | | | | | | | | | | | | | | |
| **True Conc. (mg/Kg)** | **R1** | | **R2** | | **R3** | | **R4** | | **R5** | | **R6** | | **R7** | | **R8** | | **R9** | | **R10** | | **Mean** | | **SD** | **% RSD** | | **% Deviation** |
| 0.1 | 0.105 | | 0.124 | | 0.122 | | 0.123 | | 0.124 | | 0.100 | |  | |  | |  | |  | | 0.116 | | 0.011 | 9.15 | | -16.38 |
| 0.2 | 0.190 | | 0.199 | | 0.186 | | 0.192 | | 0.180 | | 0.185 | |  | |  | |  | |  | | 0.189 | | 0.006 | 3.41 | | 5.69 |
| 0.5 | 0.530 | | 0.538 | | 0.511 | | 0.540 | | 0.547 | | 0.548 | | 0.475 | | 0.547 | | 0.467 | | 0.466 | | 0.517 | | 0.014 | 2.68 | | -3.41 |
| 1 | 1.030 | | 1.057 | | 0.956 | | 0.994 | | 1.000 | | 1.000 | |  | |  | |  | |  | | 1.006 | | 0.034 | 3.43 | | -0.62 |
| 2 | 1.947 | | 1.927 | | 1.941 | | 1.952 | | 1.952 | | 2.028 | |  | |  | |  | |  | | 1.958 | | 0.036 | 1.82 | | 2.11 |
| 5 | 4.825 | | 5.128 | | 5.076 | | 5.097 | | 5.067 | | 4.892 | |  | |  | |  | |  | | 5.014 | | 0.124 | 2.48 | | -0.28 |

**Matrix Effect** = (Slope of Matrix Curve-Slope of Solvent Cure)/ Slope of Solvent Curve*100 ME % = 1.88

**Calibration curve of Thiamethoxam in matrix (Okra)**

**Specificity of Thiamethoxam**

Chromatogram of control sample in okra with Thiamethoxam (Rt = 5.93 min)

Result: The excipient compound do not interfere with the analysis of the targeted analyte Azoxystrobin

**Repeatability**

| **Replicates** | **Original Conc. (mg/Kg)** | **Obtained Retention Time (Min)** | **Obtained Peak Area (mAU*min)** | **Calculate Conc. (mg/Kg) from Peak area** |
| --- | --- | --- | --- | --- |
| **R1** | 0.5 | 5.96 | 1.253 | 0.53 |
| **R2** | 0.5 | 5.95 | 1.27 | 0.538 |
| **R3** | 0.5 | 5.95 | 1.207 | 0.511 |
| **R4** | 0.5 | 5.94 | 1.276 | 0.54 |
| **R5** | 0.5 | 5.94 | 1.292 | 0.547 |
| **R6** | 0.5 | 5.93 | 1.294 | 0.548 |
| **R7** | 0.5 | 5.93 | 1.124 | 0.475 |
| **R8** | 0.5 | 5.92 | 1.292 | 0.547 |
| **R9** | 0.5 | 5.91 | 1.106 | 0.467 |
| **R10** | 0.5 | 5.91 | 1.103 | 0.466 |
| **Mean** |  | 5.93 | 1.222 | 0.517 |
| **SD** |  | 0.016 | 0.08 | 0.014 |
| **% RSD** |  | 0.274 | 6.27 | 2.68 |

**Limit of Detection (LOD) and Limit of Quantification (LOQ)**

| **LOD =** 3.3 x Residual standard deviation (STEYX) / Slope | | | | |
| --- | --- | --- | --- | --- |
| **LOQ =** 10 x Residual standard deviation (STEYX) / Slope | | | | |
| **STEYX =** Standard Error of the estimate Y on X through excel | | | | |
| **True Conc. (mg/Kg)** | **Mean Area (mAU*min)** | **Steyx Value** | **LOD** | **LOQ** |
| 0.1 | 0.288 | 0.060 | 0.86 | 0.259  Rounded off to 0.3 mg/Kg |
| 0.2 | 0.457 |  |  |  |
| 0.5 | 1.222 |  |  |  |
| 1 | 2.361 |  |  |  |
| 2 | 4.579 |  |  |  |
| 5 | 11.70 |  |  |  |

**Recovery of Thiamethoxam**

| **Replicate** | **LOQ (0.3 mg/kg)** | | | **5 LOQ (1.5 mg/kg)** | | | **10 LOQ (3 mg/kg)** | | |
| --- | --- | --- | --- | --- | --- | --- | --- | --- | --- |
|  | **Peak Area** | **Conc (mg/Kg)** | **% Recovery** | **Peak Area** | **Conc (mg/Kg)** | **% Recovery** | **Peak Area** | **Conc (mg/Kg)** | **% Recovery** |
| **R1** | 0.563 | 0.234 | 78.11 | 3.107 | 1.326 | 88.41 | 6.365 | 2.724 | 90.82 |
| **R2** | 0.545 | 0.227 | 75.54 | 3.063 | 1.307 | 87.15 | 6.674 | 2.857 | 95.24 |
| **R3** | 0.575 | 0.239 | 79.83 | 3.014 | 1.286 | 85.75 | 6.52 | 2.791 | 93.03 |
| **R4** | 0.543 | 0.226 | 75.25 | 2.98 | 1.272 | 84.78 | 6.559 | 2.808 | 93.59 |
| **R5** | 0.563 | 0.234 | 78.11 | 2.956 | 1.261 | 84.09 | 6.321 | 2.706 | 90.19 |
| **Mean** | 0.558 | 0.232 | 77.37 | 3.024 | 1.291 | 86.04 | 6.321 | 2.706 | 90.19 |
| **SD** | 0.014 | 0.006 | 1.936 | 0.061 | 0.026 | 1.756 | 0.145 | 0.062 | 2.069 |
| **% RSD** | 2.43 | 2.50 | 2.50 | 2.03 | 2.04 | 2.04 | 2.23 | 2.24 | 2.24 |

**Percent Recovery**

| **% Recovery** = Average Concentration in sample x 100  True Concentration in sample | | | |
| --- | --- | --- | --- |
|  | **LOQ** | **5 LOQ** | **10 LOQ** |
| **% Recovery** | 77.37 | 86.04 | 90.19 |
| **% RSD** | 2.5 | 2.04 | 2.24 |

Recovery % - Within the recommended range of 70-120% with RSD <20%

**Reproducibility of Thiamethoxam**

| **Within Laboratory** | | | | | | | | | |
| --- | --- | --- | --- | --- | --- | --- | --- | --- | --- |
| **Date of Analysis: 9.1.2024** | | | | | | | | | |
| **True Conc. (mg/Kg)** | **Peak Area (mAU*min)** | | | | | | **Mean** | **SD** | **% RSD** |
|  | **R1** | **R2** | **R3** | **R4** | **R5** | **R6** |  |  |  |
| **2** | 2.418 | 2.480 | 2.244 | 2.334 | 2.346 | 2.346 | 2.361 | 0.080 | 3.40 |
| 5 | 4.553 | 4.507 | 4.54 | 4.565 | 4.564 | 4.742 | 4.579 | 0.083 | 1.81 |
|  | | | | | | | | | |
| **Date of Analysis: 23.3.2024** | | | | | | | | | |
| **True Conc. (mg/Kg)** | **Peak Area (mAU*min)** | | | | | | **Mean** | **SD** | **% RSD** |
|  | **R1** | **R2** | **R3** | **R4** | **R5** | **R6** |  |  |  |
| **2** | 2.162 | 2.14 | 2.25 | 2.172 | 2.225 | 2.198 | 2.191 | 0.041 | 1.88 |
| **5** | 4.211 | 4.297 | 4.264 | 4.27 | 4.252 | 4.195 | 4.248 | 0.038 | 0.90 |

Reproducibility < 20%

**Robustness of Thiamethoxam**

| **Mobile phase in validated method: 60 : 40 Acetonitrile : Water** | | | | | | | | |
| --- | --- | --- | --- | --- | --- | --- | --- | --- |
| **Peak Area (mAU*min)** | | | | | | | | |
| **True Conc. (mg/Kg)** | **R1** | **R2** | **R3** | **R4** | **R5** | **Mean** | **SD** | **% RSD** |
| **2** | 4.553 | 4.507 | 4.54 | 4.565 | 4.564 | 4.546 | 0.024 | 0.53 |
|  | | | | | | | | |
| **Mobile phase in Changed method: 65 : 35 Acetonitrile : Water** | | | | | | | | |
| **True Conc. (mg/Kg)** | **R1** | **R2** | **R3** | **R4** | **R5** | **Mean** | **SD** | **% RSD** |
| 2 | 4.294 | 4.2 | 4.227 | 4.212 | 4.19 | 4.225 | 0.041 | 0.97 |

| **Wavelength in validated method: 254 nm** | | | | | | | | |
| --- | --- | --- | --- | --- | --- | --- | --- | --- |
| **Peak Area (mAU*min)** | | | | | | | | |
| **True Conc. (mg/Kg)** | **R1** | **R2** | **R3** | **R4** | **R5** | **Mean** | **SD** | **% RSD** |
| 2 | 4.553 | 4.507 | 4.54 | 4.565 | 4.564 | 4.546 | 0.024 | 0.53 |
|  | | | | | | | | |
| **Wavelength in Changed method: 260 nm** | | | | | | | | |
| **True Conc. (mg/Kg)** | **R1** | **R2** | **R3** | **R4** | **R5** | **Mean** | **SD** | **% RSD** |
| 2 | 4.122 | 4.018 | 4.157 | 4.083 | 4.19 | 4.12 | 0.067 | 1.62 |

Robustness < 20%

**Uncertainty measurement of Thiamethoxam in Okra**

Test parameter Thiamethoxam Product : Okra Date of testing : 9.1.2024

Equipment Machine used for measurement HPLC-UV Temp. (ºC) : 25±5

RH (%) : 30-70

| S.NO. | Equip/Reference Material | Parameter | Measured at | Unit | U# | Unit |
| --- | --- | --- | --- | --- | --- | --- |
| 1 | CRM- Thiamethoxam | Purity | 99.46 | % | 0.8 | % |
| 2 | Analytical Balance | Weight | 10 | mg | 0.5 | mg |
| 3 | Analytical Balance | weight | 10 | gm | 0.3 | gm |
| 4 | Volumetric Flask | volume | 10 | ml | 0.01 | ml |
| 5 | Micropipette | volume | 100 | µl | 0.1 | µl |
| 6 | Micropipette | volume | 1000 | µl | 0.1 | µl |
| 7 | Recovery | Concentration | 86.036 | % | 0.785 | % |
| 8 | Linearity | Coefficient of determination |  |  | 0.999 |  |

CALCULATIONS

| Uncert  U | Source of uncertainty | Type | Observations | | Unit | Deviation  (Ai-Ᾱ) | Unit | (Ai-A)^2^ | Std. deviation Unit |
| --- | --- | --- | --- | --- | --- | --- | --- | --- | --- |
| u 1 | Repeatability | A | A1= | 0.53 | mg/kg | 0.013 | mg/kg | 0.000172 | \| Std. deviation σ \| \| Unit \| \| --- \| --- \| --- \| \| = \| √∑(Ai-Ᾱ)² \|  \| \| √ n-1 \|  \| \| = \| 0.035 \| mg/Kg \| \| Std Uncertainty u1 \| \|  \| \| = \| σ/ √n \|  \| \| = \| 0.01095 \| mg/Kg \| \|  \|  \|  \| \| Relative u1 \| \|  \| \| = \| 0.00385/0.4778 \| \| \| = \| 0.02118 \|  \| |
|  |  |  | A2= | 0.538 | mg/kg | 0.021 | mg/kg | 0.000445 |  |
|  |  |  | A3= | 0.511 | mg/kg | -0.006 | mg/kg | 3.48E-05 |  |
|  |  |  | A4= | 0.54 | mg/kg | 0.023 | mg/kg | 0.000534 |  |
|  |  |  | A5= | 0.547 | mg/kg | 0.030 | mg/kg | 0.000906 |  |
|  |  |  | A6= | 0.548 | mg/kg | 0.031 | mg/kg | 0.000967 |  |
|  |  |  | A7= | 0.475 | mg/kg | -0.042 | mg/kg | 0.0001 |  |
|  |  |  | A8= | 0.547 | mg/kg | 0.030 | mg/kg | 0.000906 |  |
|  |  |  | A9= | 0.467 | mg/kg | -0.050 | mg/kg | 0.0064 |  |
|  |  |  | A10= | 0.466 | mg/kg | -0.051 | mg/kg | 0.002591 |  |
|  |  |  | Mean = 0.517  N= 10 | | mg/kg |  | | ∑ = 0.0131 |  |
| u 2 | CRM- Thiamethoxam | B | Purity 99.46% | | | Standard uncertainty  ±Uc from Cal. Cert.  = 1.162  At k=2 | | u 2 / 2 =   0.581% | Relative u 2  = 0.00584 |
| u 3 | Analytical Balance | B | Weight 10 mg | | | Standard uncertainty  ±Uc from Cal. Cert.  = 0.5 mg  At k= 2 | | u 3 / 2 =  0.025 mg | Relative u 3  = 0.02500 |
| u 4 | Analytical Balance | B | Weight 10 gram | | | Standard uncertainty  ±Uc from Cal. Cert.  = 0.3 gm  At k=2 | | u 4 / 2 =  0.15 gm | Relative u 4  = 0.001500 |
| u 5 | Volumetric flask | B | Volume 10 ml | | | Standard uncertainty  ±Uc from Cal. Cert.  = 0.01 ml  At k= 2 | | u 5 / 2 =  0.005 ml | Relative u 5  = 0.00050 |
| u 6 | Micropipette | B | Volume 100 µl | | | Standard uncertainty  ±Uc from Cal. Cert.  = 0.1µl  At k= 2 | | u 6 / 2 =  0.05 µl | Relative u 6  = 0.00050 |
| u 7 | Micropipette | B | Volume 1000 µl | | | Standard uncertainty  ±Uc from Cal. Cert.  = 0.1µl  At k= 2 | | u 7 / 2 =  0.05 µl | Relative u 7  = 0.00005 |
| u 8 | Recovery | B | Concentration 86.036 % | | | Standard uncertainty  ±Uc from recovery.  = 0.785%  At k= 2 | | u 8 / √3 =  0.45 % | Relative u 8  = 0.0053 |
| u 9 | Linearity | B | Coefficient of variation = 1 | | | Standard uncertainty  ±Uc from linearity curve  = 0.001  At k= 2 | | u 9 / √3 =  0.000577 | Relative u 9  = 0.0006 |

**Uncertainty Budget**

| Uncert | Source of Uncertainty | Estimate Value | Limits | Type | Distribution | F | Std. Uncertainty | Sensi-tivity co-efficient | Uncertainty contribution | D O F |
| --- | --- | --- | --- | --- | --- | --- | --- | --- | --- | --- |
| u 1 | Repeatability | 0.035 | 0.01732 | A | Normal | √10 | 0.01095413 | 1 | 0.02118 | 9 |
| u 2 | CRM- Thiamethoxam | 1.162 | 0.581 | B | Normal | 2 | 0.00584 | 1 | 0.00584 | ∞ |
| u 3 | Analytical Balance | 0.5 | 0.25 | B | Normal | 2 | 0.025 | 1 | 0.025 | ∞ |
| u 4 | Analytical Balance | 0.3 | 0.15 | B | Normal | 2 | 0.015 | 1 | 0.015 | ∞ |
| u 5 | Volumetric Flask | 0.01 | 0.005 | B | Normal | 2 | 0.0005 | 1 | 0.0005 | ∞ |
| u 6 | Micropipette | 0.1 | 0.05 | B | Normal | 2 | 0.0005 | 1 | 0.0005 | ∞ |
| u 7 | Micropipette | 0.1 | 0.05 | B | Normal | 2 | 0.00005 | 1 | 0.00005 | ∞ |
| u 8 | Recovery | 0.784956 | 0.392478 | B | Rectangular | √3 | 0.0053 | 1 | 0.0053 | ∞ |
| u 9 | Linearity | 0.001 | 0.0005 | B | Rectangular | √3 | 0.0006 | 1 | 0.0006 | ∞ |

Combined Rel. Std. Uc = √{(u1)² + (u2)² + (u3)² + (u4)² + (u5)² + (u6)² + (u7)² + (u8)² + (u9)²}

Uc = √{(0.02118)² + (0.00584)² + (0.025)² + (0.015)² + (0.0005)² + (0.0005)² + (0.00005)² + (0.0053)² + (0.0006)²}

Uc = 0.037


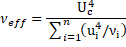


Degree of Freedom (DOF) =

=(0.037^4)/{(0.02118^4/9)+(0.00584^4/∞)+(0.025^4/∞)+(0.015^4/∞)+(0.0005^4/∞)+(0.0005^4/∞)+(0.00005^4/∞)+(0.0053^4/∞)+(0.0006^4/∞)}

= (0.037^4)/((0.02118^4)/9)= 83.8

Coverage factor k at 95% Confidence Level = 1.99 (from student t table for DoF)

**Expanded Uncertainty for mean 0.517 mg/kg, UM = 0.037 x 1.99 x 0.517 = 0.038 mg/kg**
