# Supplementary material for: Method validation and measurement uncertainty estimation of pesticide residues in Okra by GC/HPLC
Source: PLoS One. 2025 Sep 11;20(9):e0330736. doi: 10.1371/journal.pone.0330736 (PMC12425206; doi:10.1371/journal.pone.0330736)
Supplement: Attachment — Submitted filename: Clarification to queries.docx [file pone.0330736.s004.docx]

**Clarification on queries paper entitled, “Method validation and Measurement uncertainty estimation of diverse group of Pesticide residues in Okra by GC/HPLC”**

| **Journal requirements Queries** | | **Clarification** |
| --- | --- | --- |
| 1. Please ensure that your manuscript meets PLOS ONE's style requirements, including those for file naming. 2. We noticed you have some minor occurrence of overlapping text with the following previous publication(s), which needs to be addressed   In your revision ensure you cite all your sources (including your own works), and quote or rephrase any duplicated text outside the methods section.   3. We note that you have provided funding information that is not currently declared in your Funding Statement. However, funding information should not appear in the Acknowledgments section or other areas of your manuscript. 4. We strongly recommend all authors decide on a data sharing plan before acceptance. | | 1. The ms. has been modified as per PLOS ONE’s style. 2. The overlapping text with the previous publication(s), on QC chart has been addressed.   All sources including my own have been cited and the duplicate text has been rephrased.   3. The funding-related text from the acknowledgement section of the manuscript has been deleted.   The funding statement has been mentioned in the cover letter.   4. All authors have accepted to allow the data sharing. |
| **Reviewer no.** | **Query** | **Clarification** |
| **Reviewer no. 1** |  |  |
| **(i)** | With LOQs of about 0.25 mg/kg, these methods cannot reach the MRLs defined e.g. by the European Commission. | The MRLs for okra as defined by Food Safety and Standards Authority India (FSSAI) for Contaminants, Toxins and Pesticide Residues, as per Regulations, 2011 are Thiamethoxam (0.5 mg/kg), Ethion (1mg/kg), and for lambda Cyhalothrin is 2mg/kg. Hence the pesticides whose MRLs in okra were found to be above the LOQ were selected for method validation. |
| **(ii)** | There was no application to real samples to evaluate the applicability of the methods and the proportion of contaminated samples on a selection of plants. | a) The study was performed to determine the extent of compliance of validation parameters as per SANTE guidelines and not monitoring of pesticide residues in okra. Thus the work on contaminated real-world okra samples has not been reported.   However, the validated method has been tested on real samples too and can be successfully applied to real samples as well. |
| **(iii)** | Line 56 : « to control » | Corrected |
| **(iv)** | Line 58 : « may pose » | Corrected |
| **(v)** | Line 59 : « pesticide mixtures » | Corrected |
| **(vi)** | Line 69 : « precision » is mentioned twice | Corrected |
| **(vii)** | Line 93: “neonicotinoids | Corrected |
| **(viii)** | Line 113: make it clearer that you used an extraction protocol of yours which had been published earlier. | Made clear statement regarding the extraction protocol |
| **(ix)** | Figure 4: I cannot see the line for the observed values in the plot at 1 mg/kg. | The corrected figure 4 of QC charts at LOQ of Ethion, lambda Cyhalothrin and Thiamethoxam have been incorporated in the figure file |
| **(x)** | They are several grammatical and spelling mistakes in particular in the abstract and introduction. | The grammatical and spelling mistakes have been corrected in the ms. |
| **Reviewer no. 2** |  |  |
| (i) | Capitalization inconsistency in the title | The inconsistency has been fixed as suggested |
| (ii) | **Abstract issues**   a) The introduction lacks context on why pesticide detection in okra is significant. Phrasing issues: e.g., “logical specificity” is unclear. b) The abstract lacks clear context on the problem being addressed. | a)The novel aspect regarding method validation in okra alongwith significance of monitoring pesticide residues in okra have been addressed in the abstract.   b) The relevance of determining pesticide residues in public health and food regulation has also been mentioned. |
| (iii) | **Introduction issues**   a) Redundant mentions of pesticide contamination and vague claims (e.g., "people apply pesticides mixture"). b) Redundant mention of ISO/IEC 17025 uncertainty requirements (lines 70–80). c) Use of outdated or non-specific references (e.g., Pestology 1984 [2]). d) Improve citation base with more recent references from 2015–2024. | a) Since okra is cultivated during summer and rainy seasons which are conducive for insect infestation, farmers commonly apply mixture of insecticides for crop protection. Statement has been revised. b) The sentence has been deleted c) The reference has been removed d) Recent references have been added. |
| (iv) | **Material and Methods issues**   a) Lack of justification for pesticide selection beyond availability. b) Inconsistent units (e.g., “mg/Kg” vs. “mg/kg”), overly condensed chromatography conditions   **Validation parameters**  c)The correlation coefficients (R²) and regression equations are present but lack residual analysis. No comparative method or matrix challenge was mentioned.  **Measurement Uncertainty**  d)Clarify the meaning of “u1–u7” terms  e)Elaborate on why lower values validate reliability in vegetables. | a) Justification for pesticide selection with reference is given in Introduction  b) Necessary corrections regarding inconsistent units have been done in the ms. For the purpose of comparison overlaid chromatograms have been given in figure 2.   c) Residual analysis and comparison of standard methods for these pesticides in tomatoes and cucumbers has been done in Results and Discussion   d)The meaning of u1-u7 has been elaborated  e) Elaborated why lower values of MU validate reliability in vegetables |
| (v) | **Result and Discussion issues**   a) The discussion does not critically compare GC vs. LC methods or address cross-matrix reproducibility. b) References like “(15)” or “(Fig. 2 (i-iii))” are not adequately contextualized. The discussion lacks depth on method comparison or implications of the findings.   **Figures and Tables**   c) Figures referenced (e.g., Fig. 1, 2, 3, 4) are not embedded in the main text. This reduces comprehension and violates PLOS ONE’s formatting standards.   **Conclusion** d)Summarizes the work adequately but contains redundancy with abstract and results  **References**  e) Duplicates found (Shrestha 2024 appears twice); fix citation style inconsistencies (some lack DOIs).  f)The citation style is inconsistent and lacks uniform formatting | a) Comparison of GC vs. LC methods or address cross-matrix reproducibility has been done in the text. b) A comparative paragraph on how this method performs versus LC-MS/MS or published QuEChERS-GC methods has been added.   c) As per PLOS ONE’s style, Figure captions must be inserted in the text of the manuscript, immediately following the paragraph in which the figure is first cited so Figures were not embedded in the text but submitted as separate files.  d)Conclusion has been modified  e)The duplicacy of Shrestha 2024 reference has been removed. DOIs of all papers cited, has been given except the ones who’s DOIs are unavailable.  f)The citation style has been made consistent as per PLOS ONE author guidelines |
| (vi) | **Questions Inferred from Manuscript** 1. Could this method be extended or adapted for detecting similar residues in other vegetables or high-water content matrices like cucumber?  2. How does this method compare detection limits and cost to advanced LC-MS/MS-based multi-residue protocols?  3. Are the measurement uncertainties consistent across different analysts or labs (interlaboratory validation potential)? | T this  1.This method can be extended for detecting similar residues in other vegetables like bean, tomatoes, chili, cabbage, cauliflower, brinjal etc. where it has been tested. The method has not been validated on high-water content matrices like cucumber etc. but probably the QuEChERS method can be validated on high-water content matrices too as such studies have been reported in cucumber.  2. The detection limits of LC-MS/MS-based multi-residue protocols are definitely very low such that they can meet the EU MRLs but the LC-MS/MS is highly expensive and is not available in our lab.  Since we have performed analysis using HPLC and GC system we could detect up to the MRLs set by Food Safety and Standards Authority India (FSSAI) for Contaminants, Toxins and Pesticide Residues, as per Regulations, 2011. The method validation parameters as per SANCO guidelines could be achieved in the process.  3. The measurement uncertainties are based upon intralaboratory validation |
| **Reviewer 3** |  |  |
| **(i)** | **a)** Lack of Supporting Data and Transparency in Calculation  A critical shortcoming of the manuscript is the absence of supporting data. Essential information and raw data underlying the method validation and uncertainty estimation are not provided. The manuscript does not explain how the measurement uncertainty (MU) was calculated, nor are the input values or assumptions documented. For a method that may be used in ISO/IEC 17025-accredited laboratories, this level of detail is imperative. A supplementary section containing raw data, calculation spreadsheets, and validation outputs would greatly enhance transparency and reproducibility. b) Lack of Real Sample Analysis and Reference Materials The study appears to rely solely on spiked matrixes without including contaminated real-world okra samples. This limits the applicability and relevance of the method to actual monitoring scenarios.   c) Matrix Considerations and Source Variability   The authors did not account for potential variability introduced by different varieties or sources of okra. Since agricultural matrices can vary significantly, especially in complex plant matrices like okra, it is essential to consider source-related effects on method performance.   d) Selectivity and Interferences   The manuscript lacks a proper assessment of selectivity. In the absence of mass spectrometric detection, structural analogs or co-eluting compounds may compromise the method's specificity. E. g, there is no mention of interference studies involving structurally similar pesticides that may be present in real samples.   e) e)Diastereomer Considerations for Lambda-Cyhalothrin f) Lambda-Cyhalothrin is known to be a mixture of diastereomers that are separable by GC. The paper does not address whether these isomers were resolved in the method, and if not, how quantification was handled. This is a critical omission, as the relative proportions of isomers can influence the accuracy of the method. g) f) Inadequate Method Description and Reproducibility Concerns The QuEChERS extraction protocol is not described in sufficient detail to ensure reproducibility. The manuscript refers to a paper that references another paper, instead of clearly describing the procedure used. A self-contained, step-by-step description is necessary for validation and replication. h) g) Robustness and Sample Preparation An insufficient robustness study was conducted, that relied solemnly on the instrumental (detection) parameters. Key aspects of sample preparation (e.g., drying, grinding, extraction, cleanup) are major contributors to variability and measurement uncertainty. The impact of these factors should have been assessed both in the robustness study and in the uncertainty estimation. i)   Minor Comments 1. Figures and Visual Data Quality Figure 1: The chemical structures lack uniformity in style; the structures of Ethion and Cyhalothrin appear distorted. Figure 2B: The observed value line is missing, making it impossible to interpret the graph properly. 2. Units The authors should use SI-compliant units such as “mg kg⁻¹” or “mg/kg,” following recommendations by IUPAC, BIPM, and other standards bodies. 3. Language and Typos The manuscript requires language polishing. Several typographical and grammatical errors are present. For example: Line 131: “Europeon [sic!] Commission” should be “European Commission” Line 69: The phrase “assays of precision, bias, linearity, precision…” is redundant and unclear. | Saa a) Raw Data will be provided in the supplementary section for transparency in calculation  Raw Data indicating calculation of measurement  uncertainty (MU) will also be provided in the  supplementary section  b)The study was performed to determine the extent of compliance of validation parameters as per SANTE guidelines and not monitoring of pesticide residues in okra. The validated method has been tested on real samples too.   c) The method validation study was undertaken on the main variety of okra (Abelmoschus esculentus (L.) which is cultivated in this region.   Though significant variation is observed in various morphological traits like plant height, pod length, number of branches, and yield in different varieties of okra but they are not known to affect the method validation parameters.   d) Due to non availability of mass spectrometric detection, pesticides were analysed singly so that there is no interference of structurally similar pesticides. Moreover, the okra vegetable taken for method validation was from the vegetable research centre (VRC) plot where no pesticide was sprayed. e) Lambda-Cyhalothrin was validated as such in the form of a single compound throughout the study. Resolution of isomers was not observed. f) The reference of the detail QuEChERS extraction protocol was already given in the text. However, a step-by-step description which is necessary for validation and replication has been added in the methodology section. g) Robustness was conducted on the instrumental parameters like change in flow rate, method programming, change in mobile phase concentration and detection wavelength, which are mainly responsible for determining the robustness of the validated method. Similar type of studies regarding robustness of the method have been reported in other method validation studies too. 1. Figure 1 The chemical structures of Ethion and Cyhalothrin have been corrected   Figure 2B The observed value line is corrected and the graph has been interpreted properly   2. Correction regarding use of units has been done 3. Typographical and grammatical errors have been corrected   Line 131: “Corrected as suggested  Line 69: The phrase “assays of precision, bias, linearity, precision has been clarified. |
| **Reviewer 4** | 1) 1) At abstract, line number 36 please modify 0.3mg/kg to be 0.30 mg/kg. 2) Please justify the alignment for the whole manuscript. 3) Please try to increase the figures resolution 4) At Introduction line 56: modify from Several groups of pesticides are used to for controlling weeds to be Several groups of pesticides are used for controlling weeds. | 1) Done as suggested  2) The alignment of the ms. has been changed to justified.  3) The figure resolution has been increased  4) Done as suggested and modified as per Reviewer 1 |
| **Reviewer 5** | 1-The selection of pesticides (Thiamethoxam, Ethion, and lambda-Cyhalothrin) is appropriate, but it would be helpful to briefly mention why these specific compounds were targeted.  2-The use of the modified QuEChERS method followed by GC/HPLC is standard and effective, though it would improve clarity if the reason for choosing both GC and HPLC (rather than one) is explained.  3.The abstract contain the reported validation parameters that are acceptable (e.g., R² > 0.99, recovery > 70%, RSD < 20%), but mentioning the actual number of pesticides tested would add value. 4. Some sentences are overly long and would benefit from simplification.  5.Minor grammatical issues exist, such as: "method validation were tested" → should be "was tested". "diverse group in okra matrix" → better phrased as "a diverse group of pesticide residues in the okra matrix".  6.The abstract does not clearly state what is novel or unique about this study compared to previous similar work. This should be addressed in one sentence.  7. Although the method is described as "simple, rapid, and cost-effective", no greenness assessment has been included to support its environmental friendliness. Given the increasing emphasis on green analytical chemistry, it is strongly recommended to evaluate the method’s greenness using one or more established tools such as the Analytical Eco-Scale, AGREE, or GAPI. This will enhance the relevance and sustainability value of the proposed method. | 1. The reason for selecting the specific compounds Thiamethoxam, Ethion, and lambda-Cyhalothrin has been elaborated in the Introduction.  2. All the three pesticides were analysed individually. HPLC-UV was used for analysis of Thiamethoxam while Ethion, and lambda-Cyhalothrin were analysed using GC-ECD .  3.Actual number of pesticides has been mentioned in abstract.   4. 4.Long sentences have been shortened in the text. 5.   5.Done as suggested   6. Abstract has been modified 7. 7.Greenness assessment through Analytical Eco Scale (AES) has been included in introduction and result obtained through AES in results section. The whole procedure of extraction and analysis was carried out following good agricultural practices (GAP) and mild solvents which were less toxic. |
| **Reviewer 6** | This MS developed an analytical method for measuring three pesticides in Okra. And this is a very comment method used for analyzing pesiticides, and there are well documented publications on this old topic, this study did not explain why they did this work and the current status of the analytical method for these pesticides. overall, this study lacks of novelty | Thiamethoxam, Ethion, and lambda-Cyhalothrin are three insecticides that are applied on okra crop in this subtropical region. This study was undertaken to develop and evaluate a cost effective and reliable method that can provide results with high accuracy using simple equipments like HPLC and GC instead of highly expensive GC/LC-MS which are not available in all the labs. |
