# Supplementary material for: Method validation and measurement uncertainty estimation of pesticide residues in Okra by GC/HPLC
Source: PLoS One. 2025 Sep 11;20(9):e0330736. doi: 10.1371/journal.pone.0330736 (PMC12425206; doi:10.1371/journal.pone.0330736)
Supplement: Attachment — Submitted filename: Response to reviewers.docx [file pone.0330736.s006.docx]

**Response to the academic editor and reviewers on paper entitled, “Method validation and Measurement uncertainty estimation of diverse group of Pesticide residues in Okra by GC/HPLC”**

| **S. No** | **Comments to the Author** | **Reviewer no.** | **Response from reviewer** | **Clarification** |
| --- | --- | --- | --- | --- |
| 1. | If the authors have adequately addressed your comments raised in a previous round of review and you feel that this manuscript is now acceptable for publication, you may indicate that here to bypass the “Comments to the Author” section, enter your conflict of interest statement in the “Confidential to Editor” section, and submit your "Accept" recommendation. | # 1 | No comments | No clarification required |
|  |  | # 4 | All comments have been addressed | No clarification required |
|  |  | # 5 | All comments have been addressed | No clarification required |
| 2. | Is the manuscript technically sound, and do the data support the conclusions? | # 1 | Yes | No clarification required |
|  |  | # 4 | Yes | No clarification required |
|  |  | # 5 | Yes | No clarification required |
| 3. | Has the statistical analysis been performed appropriately and rigorously? | # 1 | Yes | No clarification required |
|  |  | # 4 | Yes | No clarification required |
|  |  | # 5 | Yes | No clarification required |
| 4. | Have the authors made all data underlying the findings in their manuscript fully available? | # 1 | Yes | No clarification required |
|  |  | # 4 | Yes | No clarification required |
|  |  | # 5 | Yes | No clarification required |
| 5. | Is the manuscript presented in an intelligible fashion and written in standard English? | # 1 | Yes | No clarification required |
|  |  | # 4 | Yes | No clarification required |
|  |  | # 5 | Yes | No clarification required |
| 6. | Review Comments to the Author | # 1 | The authors have addressed my comments relatively adequatetly. Yet, I am still a little frustrated not to see any data on real samples, in particular since the authors mentioned that they have some. Unless they have strong arguments not to publish these data in the present paper, I strongly recommend they do so. Even if the data are intended for another paper, a small subset could be published along with this methodological paper to illustrate the applicability of the method and reinforce the paper. | The real samples of okra are laced with a number of different pesticides which could interfere while method validation of these pesticides. Since the present study was specifically conducted to validate a method for estimation of the three commonly applied pesticides the data on real samples was not much generated. |
|  |  | # 4 | I sincerely appreciate the editor's suggestion that I revise this manuscript. After the second revision, I suggest that the manuscript can be accepted in its current form. | Clarification has been given and an explanation regarding the query has also been given in the revised ms. |
|  |  | # 5 | The authors have revised and answered all the questions. The current revised manuscript can be published in PLOS One journal. | No clarification required |
| 7. | PLOS authors have the option to publish the peer review history of their article ([what does this mean?](https://journals.plos.org/plosone/s/editorial-and-peer-review-process#loc-peer-review-history)). If published, this will include your full peer review and any attached files. | # 1 | Yes | - |
|  |  | # 4 | No | - |
|  |  | # 5 | Yes | - |
