# Supplementary material for: Method validation and measurement uncertainty estimation of pesticide residues in Okra by GC/HPLC
Source: PLoS One. 2025 Sep 11;20(9):e0330736. doi: 10.1371/journal.pone.0330736 (PMC12425206; doi:10.1371/journal.pone.0330736)
Supplement: Attachment — Submitted filename: Reviewer Comments.doc [file pone.0330736.s005.doc]

Manuscript Number: PONE-D-25-17690R1

Method validation and Measurement uncertainty estimation of diverse group of Pesticide residues in Okra by GC/HPLC

**Mohamed A. Elsayed**

Lecturer of Pharmaceutical Analytical Chemistry, Faculty of Pharmacy, Fayoum University, Fayoum 63514, Egypt,

ORCID ID: 0000-0003-2673-3301

Reviewer comments:
I sincerely appreciate the editor's suggestion that I revise this manuscript.
After the second revision, I suggest that the manuscript can be accepted in its current form.
